# Effectiveness of caudal septal extension graft application in endonasal septoplasty^☆^

**DOI:** 10.1016/j.bjorl.2016.01.014

**Published:** 2016-04-20

**Authors:** Yunus Karadavut, Ilker Akyıldız, Hatice Karadaş, Aykut Erdem Dinç, Gökçe Tulacı, Eren Tastan

**Affiliations:** a Ankara Training and Research Hospital ENT Clinic, Ankara, Turkey; b Bulent Ecevit University, ENT Clinic, Zonguldak, Turkey

**Keywords:** Nasal septum, Nasal cartilage, Nasal obstruction, Intranasal surgery, Grafting, Septo nasal, Cartilagem nasal, Obstrução nasal, Cirurgia intranasal, Aplicação de enxerto

## Abstract

**Introduction:**

Septal deviation is a common disease seen in daily otorhinolaryngology practice and septoplasty is a commonly performed surgical procedure. Caudal septum deviation is also a challenging pathology for ear, nose, and throat specialists. Many techniques are defined for caudal septal deviation.

**Objective:**

To evaluate the effectiveness of caudal septal extension graft (CSEG) application in patients who underwent endonasal septoplasty for a short and deviated nasal septum.

**Methods:**

Forty patients with nasal septal deviation, short nasal septum, and weak nasal tip support who underwent endonasal septoplasty with or without CSEG placement between August 2012 and June 2013 were enrolled in this study. Twenty patients underwent endonasal septoplasty with CSEG placement. The rest of the group, who rejected auricular or costal cartilage harvest for CSEG placement, underwent only endonasal septoplasty without any additional intervention. Using the Nasal Obstruction Symptom Evaluation (NOSE) and Rhinoplasty Outcome Evaluation (ROE) questionnaires, pre- and post-operative acoustic rhinometer measurements were evaluated to assess the effect of CESG placement on nasal obstruction.

**Results:**

In the control group, preoperative and postoperative minimal cross-sectional areas (MCA1) were 0.44 ± 0.10 cm^2^ and 0.60 ± 0.11 cm^2^, respectively (*p* < 0.001). In the study group, pre- and postoperative MCA1 values were 0.45 ± 0.16 cm^2^ and 0.67 ± 0.16 cm^2^, respectively (*p* < 0.01). In the control group, the nasal cavity volume (VOL1) value was 1.71 ± 0.21 mL preoperatively and 1.94 ± 0.17 mL postoperatively (*p* < 0.001). In the study group, pre- and postoperative VOL1s were 1.72 ± 0.15 mL and 1.97 ± 0.12 mL, respectively (*p* < 0.001). Statistical analysis of postoperative MCA1 and VOL1 values in the study and the control groups could not detect any significant intergroup difference (*p* = 0.093 and 0.432, respectively). In the study group, mean nasolabial angles were 78.15 ± 4.26° and 90.70 ± 2.38°, respectively (*p* < 0.001).

**Conclusion:**

Endonasal septoplasty with CESG placement is an effective surgical procedure with minimal complication rate for subjects who have a deviated, short nasal septum and weak nasal tip support.

## Resumo

**Introdução:**

Desvio septal é doença comum no cotidiano da prática otorrinolaringológica, e a septoplastia é procedimento cirúrgico comumente realizado. Desvio caudal do septo nasal é também uma condição desafiadora para os otorrinolaringologistas. São muitas as técnicas definidas para desvio caudal do septo nasal.

**Objetivo:**

Avaliar a eficácia da aplicação de enxerto de extensão septal caudal (EESC) em pacientes que passaram por septoplastia endonasal devido a septo nasal curto e com desvio.

**Método:**

Foram recrutados para o estudo, 40 pacientes com desvio de septo nasal, septo nasal curto e fraca sustentação da ponta do nariz, tratados com septoplastia endonasal com ou sem a aplicação de EESC, entre agosto de 2012 e junho de 2013. Ao todo, 20 pacientes foram tratados com septoplastia endonasal com aplicação de EESC. O restante do grupo, que rejeitou coleta de cartilagem auricular ou costal para a aplicação de EESC, foi tratado apenas com septoplastia endonasal, sem qualquer outra intervenção. Com a aplicação dos questionários NOSE (*Nasal Obstruction Symptom Evaluation*, Avaliação dos Sintomas de Obstrução Nasal) e ROE (*Rhinoplasty Outcome Evaluation*, Avaliação dos Desfechos da Rinoplastia), as mensurações pré e pós-operatórias com o rinômetro acústico foram obtidas com o objetivo de avaliar o efeito da aplicação de EESC na obstrução nasal.

**Resultados:**

No grupo controle, as áreas de secção transversal mínima (ASTM1) antes e depois da operação foram 0,44 ± 0,10 cm^2^ e 0,60 ± 0,11 cm^2^, respectivamente (*p* < 0,001). No grupo de estudo, os valores antes e depois da operação para ASTM1 foram 0,45 ± 0,16 cm^2^ e 0,67 ± 0,16 cm^2^, respectivamente (*p* < 0,01). No grupo controle, o valor para os volumes da cavidade nasal (VOL1) foi 1,71 ± 0,21 mL no pré-operatório e 1,94 ± 0,17 mL no pós-operatório (*p* < 0,001). No grupo de estudo, os VOL1 antes e depois da operação foram 1,72 ± 0,15 mL e 1,97 ± 0,12 mL, respectivamente (*p* < 0,001). A análise estatística dos valores pós-operatórios para ASTM1 e VOL1 nos grupos de estudo e controle não permitiu a detecção de qualquer diferença intergrupos (*p* = 0,093 e 0,432, respectivamente). No grupo de estudo e no grupo controle, os ângulos nasolabiais médios foram 78,15 ± 4,26° e 90,70 ± 2,38°, respectivamente (*p* < 0,001).

**Conclusão:**

A septoplastia endonasal com aplicação de EESC é um procedimento cirúrgico efetivo, com mínimo percentual de complicações para pacientes que se apresentam com septo nasal curto e com desvio e com fraca sustentação da ponta do nariz.

## Introduction

Septal deviation of the nose is one of the most common disorders seen in daily otorhinolaryngology practice, and septoplasty is a frequently performed surgical procedure by ear, nose, and throat specialists.^1^ Despite the fact that many surgical methods have been defined, such as morselization, cross-hatching incision, partial thickness incision, swing-door flap, and cut-suture technique, no single surgical procedure is successfully applicable in all conditions.^1^, ^2^

Short septal cartilage and weak nasal tip support are frequently seen nasal pathologies in patients with nasal obstruction. Conventional septoplasty techniques are not effective as a result of cartilage memory, and open techniques are invasive and time consuming. Since this is a challenging condition and conventional techniques have been unsatisfactory, efforts have been focused on developing novel surgical techniques to overcome this problem. As one of these techniques, caudal septal extension graft (CSEG) placement to support the tip of the nose was developed by Byrd et al.^3^ However, the effectiveness of this technique has not been extensively studied before in subjects with caudal nasal septal deviation, short nasal septum, and weak nasal tip support.

The aim of the present study was to evaluate the efficacy of CSEG in patients who underwent endonasal septoplasty for a short and deviated nasal septum.

## Methods

### Study design

The study was conducted in accordance with the principles of the Helsinki Declaration and approved by the local Institutional Review Board (No. 0542, date: 26/03/2014). Medical records of 40 patients who underwent endonasal septoplasty and CSEG placement between August 2012 and June 2013 were retrospectively reviewed.

Twenty patients underwent endonasal septoplasty with CSEG placement (Study Group). The remainder of the patients, who rejected auricular or costal cartilage harvest for CSEG placement, had only endonasal septoplasty, without any additional intervention such as turbinectomy or turbinoplasty (Control Group).

**Figure 1:**
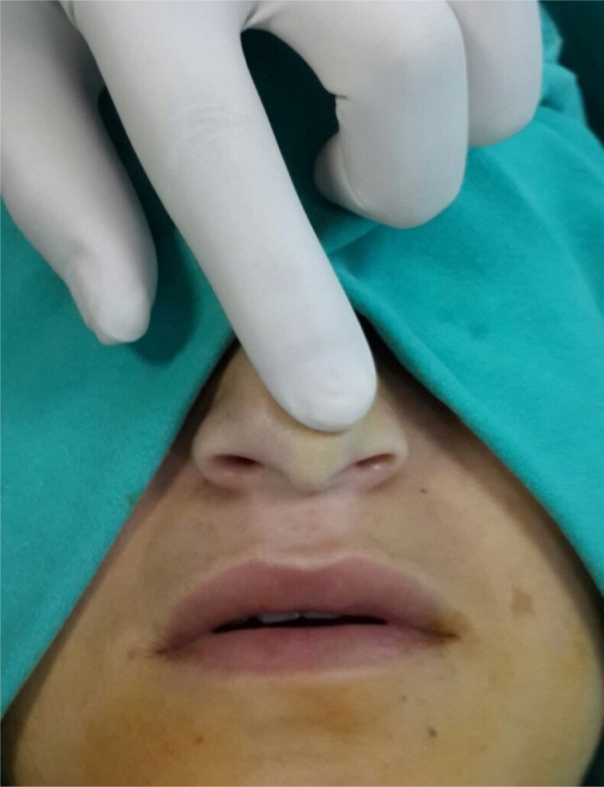
Recoil maneuver.

All patients were examined by a physician and a consultant before the surgical decision-making process. Nasal tip support was examined by recoil maneuver (Fig. 1). All subjects were evaluated by paranasal computerized tomography to reveal potential coexisting nasal or paranasal pathologies. Subjects who had mental retardation, craniofacial anomaly, active inflammatory sinonasal disease (allergic rhinitis, acute or chronic sinusitis), asthma, dorsal septal deviation, acute nasolabial angle due to long and strong lateral crus, concha bullosa, or septal perforation were excluded from the study.

### Outcome parameters

All patients were evaluated pre- and postoperatively with acoustic rhinometer with and without topical nasal decongestant (RhinoMetrics SRE2000, Interacoustics AS – DK.5610, Assens, Denmark) and they were requested to complete the Nasal Obstruction Symptom Evaluation (NOSE) (Table 1) and the Rhinoplasty Outcome Evaluation (ROE) questionnaires (Table 2) pre- and postoperatively. Pre-operatively and at eight postoperative months, minimal cross-sectional areas (MCA1) and volumes (VOL1) of the nasal cavities were measured with an acoustic rhinometer, before and after topical nasal decongestant (0.05% oxymetazoline hydrochloride) application in order to minimize nasal cycle. In both groups, pre- and postoperative MCA1 values at the deviation side (convex side) were analyzed. In both groups pre- and postoperatively, nasal volumes (VOL1) of the deviation side and the contralateral side were also evaluated before and after topical nasal decongestive application.

**Table 1:** Nasal Obstruction Symptom Evaluation (NOSE) questionnaire.

	Not a problem	Mild problem	Moderate problem	Bad problem	Severe problem
Nasal congestion or stuffiness	0	1	2	3	4
Frequency of nasal congestion	0	1	2	3	4
Trouble breathing through your nose	0	1	2	3	4
Trouble sleeping	0	1	2	3	4
Unable to get enough air through your nose during exercise or exertion	0	1	2	3	4

**Table 2:** Rhinoplasty Outcome Evaluation (ROE) questionnaire.

Do you like the external view of your nose?	0 (No)	1	2	3	4 (Yes)
How well do you breathe?	0 (Not at all)	1	2	3	4 (Very well)
Do you think your friends like your nose?	0 (Not at all)	1	2	3	4 (Always)
Does your own nose restrict your social and professional activity?	0 (Always)	1	2	3	4 (Never)
Do you think that your nose is as good as possible?	0 (Not at all)	1	2	3	4 (Yes)
Do you want to change your nose appearance and function via an operation?	0 (Absolutely)	1	2	3	4 (No)

### Surgical procedure

**Figure 2:**
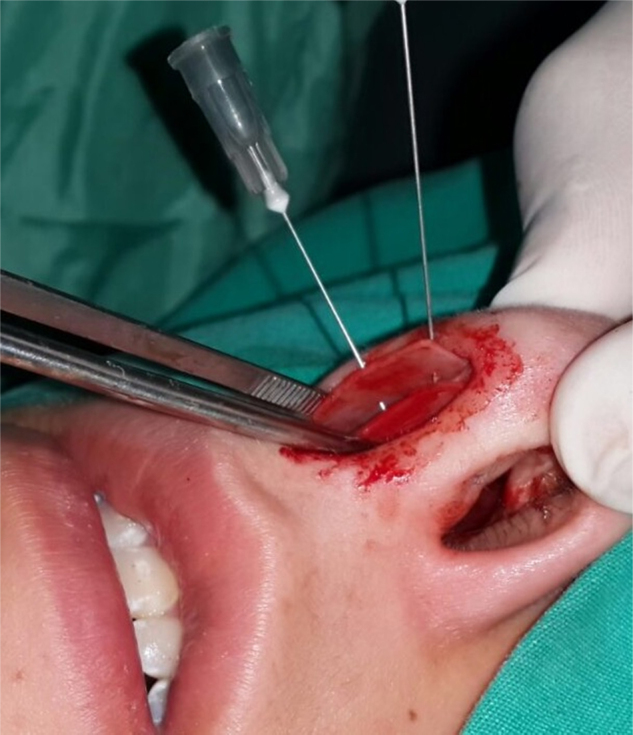
Application of caudal septal extension graft (CSEG).

**Figure 3:**
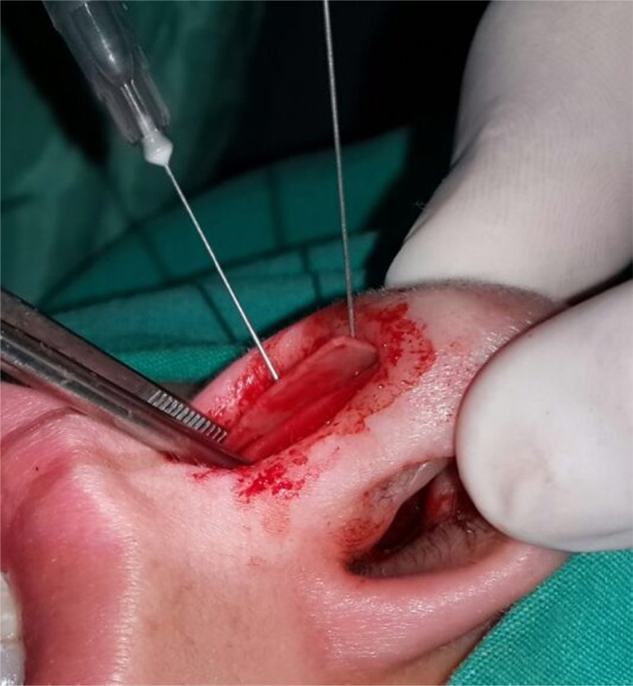
Application of caudal septal extension graft (CSEG).

**Figure 4:**
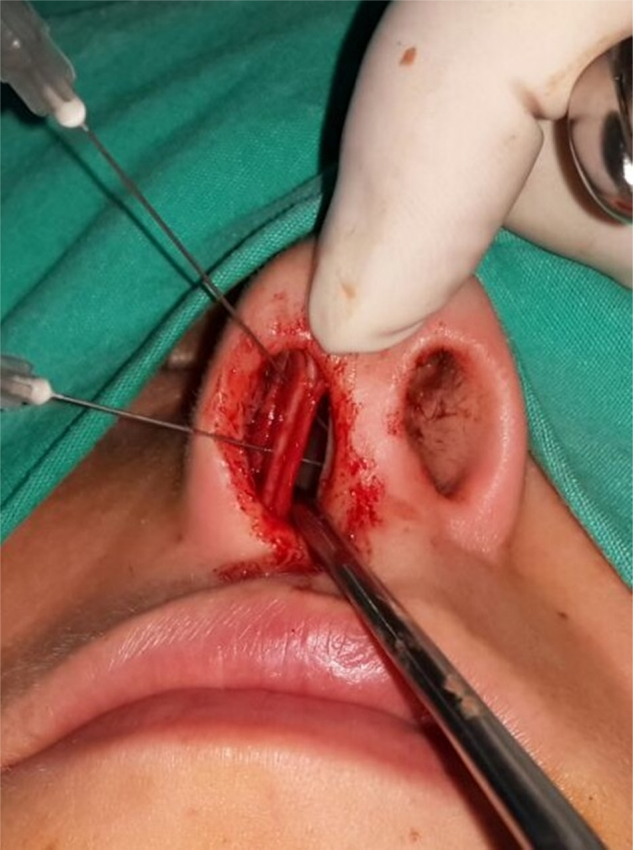
Application of caudal septal extension graft (CSEG).

Surgical procedures were performed with either local or general anesthesia, with hemitransfixion incision via endonasal approach by the same surgeon (K.Y.). All four mucoperichondrial/mucoperiosteal flaps covering four tunnels were elevated to obtain a better surgical view. After resection of the deviated part of the septal cartilage and bony septum, CSEG harvested from the septal cartilage was placed on the caudal end of the septum between the medial crura of the lower lateral cartilage and stitched with 4/0 long-lasting absorbable monofilament material (Monocryl; Figure 2, Figure 3, Figure 4). A silicon nasal splint was used for nasal packing.

Endonasal septoplasty was also performed by the same surgeon (K.Y.). After elevating all four mucoperichondrial/mucoperiosteal flaps, the deviated part of the nasal septum (bony and cartilage) was resected. After septoplasty was performed, the mucopercondrial flaps were stitched with 4/0 short-lasting absorbable monofilament material (Rapide Vicryl); as in the CSEG group, a silicon nasal splint was used for nasal packing.

### Statistical analysis

Data were analyzed using SPSS v. 21 (SPSS Inc., Chicago, IL, United States). Age distribution of the subjects in the groups was analyzed with Student's *t*-test and sex distribution analysis utilized the chi-squared test. Comparative analysis of average scores for MCA1, VOL1, and the results of the questionnaire evaluating nasal obstruction and rhinoplasty outcome as assessed by the NOSE and ROE scoring systems was carried out using the Wilcoxon test. Postoperative average MCA1 and VOL1 values in the different groups were analyzed with the Mann–Whitney U test. Changes in the nasolabial angles of the patients were measured based on lateral photographs of the patients in different groups and analyzed using Student's *t*-test for dependent groups. All differences associated with a chance probability of 0.05 or less were considered to be statistically significant.

## Results

The Study Group included 20 patients (15 males, five females) with a mean age of 31.7 ± 8.8 years (range, 23–40) and the Control Group included 20 patients (12 males, eight females) with a mean age of 34.7 ± 8.3 years (range, 26–43). Both groups did not differ from each other regarding age and gender (*p* = 0.500 and *p* = 0.281, respectively).

**Figure 5:**
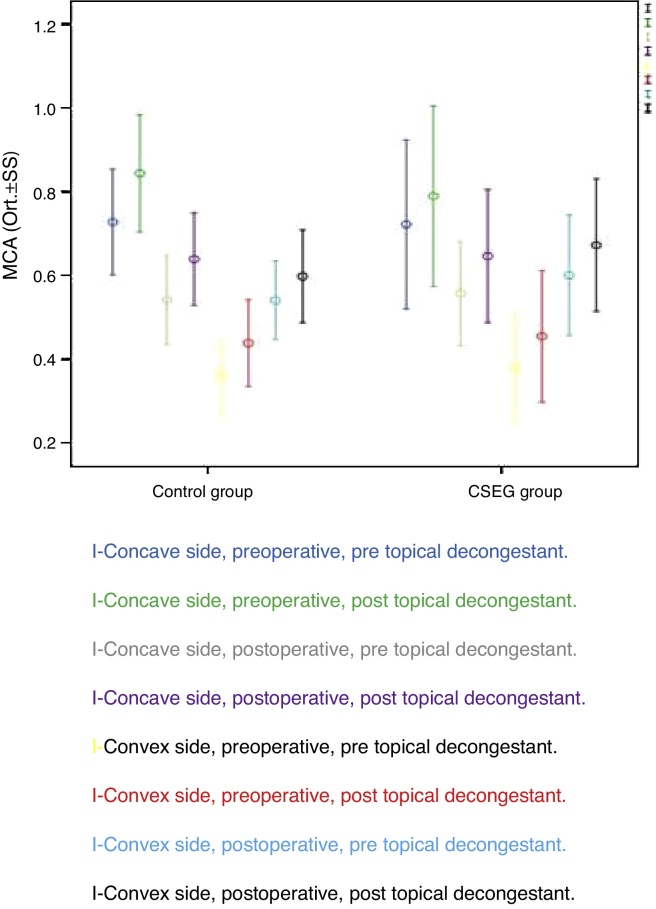
Average minimal cross-section area (MCA1) values of the study and control group.

**Figure 6:**
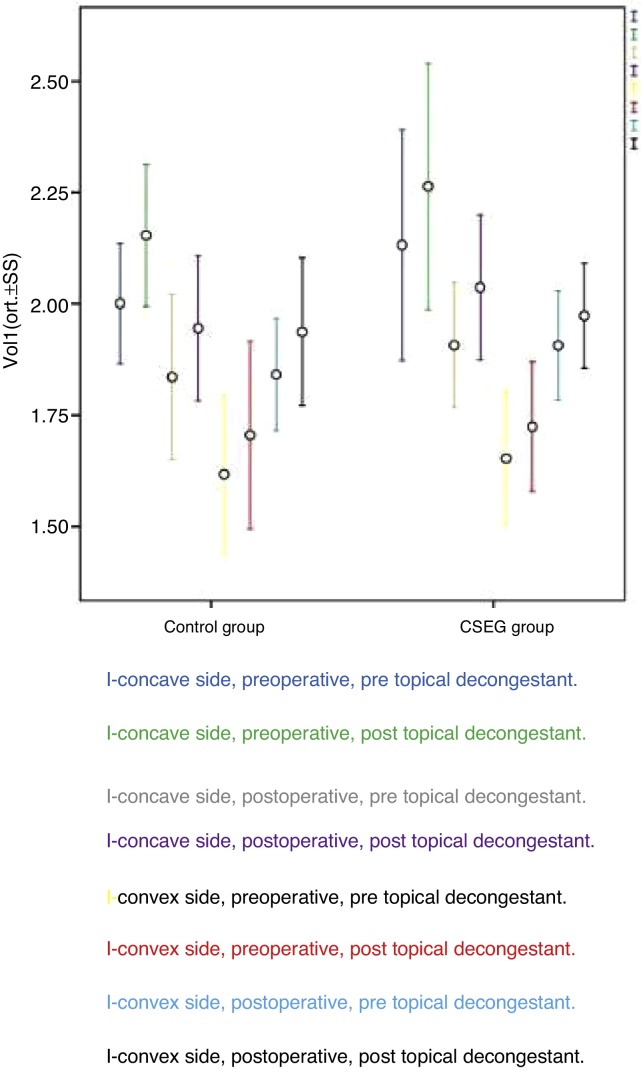
Average nasal cavity volume (VOL1) values of the study and control group.

Postoperative MCA1 values were better than preoperative MCA1 values at the deviation (convex) side in both groups (*p* < 0.001), without any statistically significant difference between Study and Control Groups (*p* = 0.093; Fig. 5). In both groups, VOL1 values were better at the deviation side after decongestion and surgery (*p* < 0.001) without any significant difference between groups (*p* = 0.432; Fig. 6).

In both groups, postoperative NOSE scale scores were better than preoperative scores (*p* < 0.001). Postoperative results were better in the Study Group when compared with the Control Group (*p* < 0.001) (Table 3). In the first, fourth, fifth, and sixth questions, postoperative results were statistically significantly better than the preoperative ones in both the Study and Control Groups (*p* = 0.049, *p* = 0.001, *p* = 0.001, and *p* = 0.038, respectively; Table 4).

**Table 3:** Nasal Obstruction Symptom Evaluation questionnaire results of the control and study group.

	Control			CSEG			Control-study comparison	
	Preop.	Postop.	*p*	Preop.	Postop.	*p*	Preop.	Postop.
Question 1	3	2	<0.001	3	0 (0–1)	<0.001	0.799	<0.001
Question 2	3	2	<0.001	3	1 (0–1)	<0.001	0.289	<0.001
Question 3	3	2	<0.001	4	0 (0–1)	<0.001	0.183	<0.001
Question 4	3	2	<0.001	3	1 (0–2)	<0.001	0.035	<0.001
Question 5	3	2	<0.001	3	1 (0–2)	<0.001	0.289	<0.001

**Table 4:** Rhinoplasty Outcome Evaluation (ROE) questionnaire results of the groups.

	Control			CSEG			Control-Study comparison	
	Preop.	Postop.	*p*	Preop.	Postop.	*p*	Preop.	Postop.
Question 1	2	2	0.046	1 (0–2)	3 (1–3)	<0.001	0.026	0.049
Question 2	1	3	<0.001	0 (0–2)	4 (3–4)	<0.001	0.165	0.602
Question 3	2	2	0.046	1 (0–3)	3 (0–3)	<0.001	0.007	0.253
Question 4	1	2	0.001	2 (0–4)	3 (2–4)	<0.001	0.063	<0.001
Question 5	1	1	0.083	2 (0–3)	3 (1–4)	<0.001	0.265	<0.001
Question 6	1	2	<0.001	2 (0–3)	3 (2–4)	<0.001	0.799	0.038

Postoperative nasolabial angle values were significantly better than the preoperative values in the study group (*p* < 0.001; Table 5).

**Table 5:** Nasolabial angles of the study and control group.

	Preop.	Postop.	*p*
Nasolabial angle of the study group (NLA)	78.15 ± 4.246	90.70 ± 2.386	<0.001
Nasolabial angle of the control group	76.254 ± 3.954	76.853 ± 4.025	>0.001

Postoperative MCA1 values were better than preoperative MCA1 values at the deviation (convex) side in both groups (*p* < 0.001), without any (statistically) significant difference between the Study and Control Groups (*p* = 0.093; Fig. 5). In both groups, VOL1 values were better at the deviation side after decongestion and surgery (*p* < 0.001), without any statistically significant difference between groups (*p* = 0.432; Fig. 6).

## Discussion

Caudal septal deviations are frequently encountered, challenging pathologies of the nose. Patients with caudal septal deviation, short nasal septum, and weak nasal tip support suffer from nasal obstruction because of deterioration of nasal airflow due to acute nasolabial angle.

Patients with acute nasolabial angle also suffer from abnormal shape of the nose because of unsatisfactory nasal tip projection.^1^, ^2^, ^4^

Satisfactory nasal tip support may be achieved with columellar strut implants placed via external approach in patients who have short nasal septa. However, in the presence of caudal septal deviation associated with short nasal septum, implantation of columellar strut alone may be insufficient. CSEG may be used to strengthen the nasal tip and correct the caudal septal deviation via endonasal incision.

Septal cartilage is a good source of CSEG, but auricular or costal cartilages may be alternative sources if septal cartilage is insufficient and the patient consents to additional incision for harvesting. Since auricular cartilage is elastic, septal and costal cartilages are considered to be superior for preparation of CSEG. In the present study, septal cartilage was preferred as first line source since it is easy to harvest.

External approach via trans-columellar incision may also be used for CSEG. In the present study, the authors preferred endonasal hemitransfixion incision because of its shorter operation time, lesser external scar tissue, lower rates of flap-related complications, and faster healing process. Long-lasting absorbable suture material instead of non-absorbable material was preferred to avoid extrusion of the suture material out of the nasal vestibular skin. Mattress stitches were used to in order to obtain a more stable nasal tip and to correct the caudal deviation of the nasal septum.

Postoperative MCA1 and VOL1 values were significantly better than the preoperative values in both the study and the control groups (*p* < 0.05). However, postoperative MCA1 and VOL1 values were significantly better in both the study and the control groups, without any significant intergroup difference (*p* > 0.05).

NOSE and ROE scale scores were better in the postoperative period in both the study and control groups (*p* < 0.05).

Significantly better outcomes were achieved in both groups, not only in laboratory evaluation (MCA1 and VOL1 measurement), but also in clinical evaluation (NOSE and ROE questionnaire), which indicated that classical septoplasty may be also an effective surgical procedure in a short and deviated nasal septum.

However, according to these results, patients who have a short, caudal septal deviation and seek a more projected nasal tip are good candidates for endoscopic implantation of CSEG.

## Conclusion

CSEG is an effective and simple surgical procedure to correct caudal septal deviations and strengthen the nasal tip support. However, if a patient with a caudal septal deviation desires to have better tip projection, CSEG may be a good alternative. Only endonasal septoplasty results in similar functional outcomes, and an additional endoscopic CSEG implantation does not improve MCA1, VOL1, and NOSE scores.

## Conflicts of interest

The authors declare no conflicts of interest.
